# MRI-based molecular imaging of epicardium-derived stromal cells (EpiSC) by peptide-mediated active targeting

**DOI:** 10.1038/s41598-020-78600-y

**Published:** 2020-12-10

**Authors:** Tamara Straub, Julia Nave, Pascal Bouvain, Mohammad Akbarzadeh, Siva Sai Krishna Dasa, Julia Kistner, Zhaoping Ding, Aseel Marzoq, Stefanie Stepanow, Katrin Becker, Julia Hesse, Karl Köhrer, Ulrich Flögel, Mohammad R. Ahmadian, Brent A. French, Jürgen Schrader, Sebastian Temme

**Affiliations:** 1 Department of Molecular Cardiology, Heinrich Heine University Düsseldorf, Düsseldorf, NRW Germany; 2 Department of Biochemistry and Molecular Biology II, Heinrich Heine University Düsseldorf, Düsseldorf, NRW Germany; 3 Biological and Medical Research Center (BMFZ), Genomics and Transcriptomics Laboratory, Heinrich Heine University Düsseldorf, Düsseldorf, NRW Germany; 4 Robert M. Berne Cardiovascular Research Center, University of Virginia, Charlottesville, VA USA; 5 Department of Biomedical Engineering, University of Virginia, Charlottesville, VA USA

**Keywords:** Cell delivery, Nanobiotechnology, Stem-cell research

## Abstract

After myocardial infarction (MI), epicardial cells reactivate their embryonic program, proliferate and migrate into the damaged tissue to differentiate into fibroblasts, endothelial cells and, if adequately stimulated, to cardiomyocytes. Targeting epicardium-derived stromal cells (EpiSC) by specific ligands might enable the direct imaging of EpiSCs after MI to better understand their biology, but also may permit the cell-specific delivery of small molecules to improve the post-MI healing process. Therefore, the aim of this study was to identify specific peptides by phage display screening to enable EpiSC specific cargo delivery by active targeting. To this end, we utilized a sequential panning of a phage library on cultured rat EpiSCs and then subtracted phage that nonspecifically bound blood immune cells. EpiSC specific phage were analyzed by deep sequencing and bioinformatics analysis to identify a total of 78 300 ± 31 900 different, EpiSC-specific, peptide insertion sequences. Flow cytometry of the five most highly abundant peptides (EP1, -2, –3, -7 or EP9) showed strong binding to EpiSCs but not to blood immune cells. The best binding properties were found for EP9 which was further studied by surface plasmon resonance (SPR). SPR revealed rapid and stable association of EpiSCs with EP9. As a negative control, THP-1 monocytes did not associate with EP9. Coupling of EP9 to perfluorocarbon nanoemulsions (PFCs) resulted in the efficient delivery of ^19^F cargo to EpiSCs and enabled their visualization by ^19^F MRI. Moreover, active targeting of EpiSCs by EP9-labelled PFCs was able to outcompete the strong phagocytic uptake of PFCs by circulating monocytes. In summary, we have identified a 7-mer peptide, (EP9) that binds to EpiSCs with high affinity and specificity. This peptide can be used to deliver small molecule cargos such as contrast agents to permit future in vivo tracking of EpiSCs by molecular imaging and to transfer small pharmaceutical molecules to modulate the biological activity of EpiSCs.

## Introduction

Epicardium derived cells (EPDCs) play a pivotal role during embryonic development of the heart, as they contribute to nearly all cardiac cell types including endothelial cells, fibroblasts and cardiomyocytes^1^. However, after birth, the epicardium enters a dormant state and covers the heart as a single-cell layer^2^. Upon myocardial infarction (MI), epicardial cells become activated, undergo endothelial to mesenchymal transition (EMT)^3^ and proliferate, leading to the generation of a multi-cell layer covering the surface of the infarcted heart^4^. The MI-induced epicardial layer can be selectively removed by enzymatic digestion^5^ and the isolated cells are termed EpiSCs (epicardium-derived stromal cells) to signify that the regeneration potential of EpiSC may be different from embryonic EPDCs^6,7^. EpiSCs secrete numerous paracrine factors that strongly promote angiogenesis and EpiSC-conditioned medium was shown to reduce infarct size^4^. EpiSCs are also known to migrate into the infarcted area of the heart where they can differentiate into multiple cell types such as endothelial cells, smooth muscle cells and fibroblasts^8^ to support the cardiac healing process^4^. Thus, the activated epicardium is a multipotent cell population with potential importance for cardiac regeneration. EpiSCs have already been shown to form new cardiomyocytes in vivo when stimulated with thymosin-β4, however, the number of newly formed cardiomyocytes is normally quite low^9^.

Two of the most important challenges facing cell-specific pharmacological interventions are that: (i) the concentration of a drug in the target tissue may be very low and (ii) the systemic application of drugs is often associated with unwanted side effects. This has been a major obstacle in cancer therapy where the systemic application of potent anti-cancer drugs can lead to severe side effects e.g. peripheral neuro- or cardiotoxicity^10^. To overcome these difficulties, drug delivery systems have been developed which enhance drug accumulation within tumor tissue by passive or active targeting. One of the most attractive actively targeted drug delivery systems uses cell-specific antibodies or peptides coupled to liposomes^11^. Targeted liposomes can encapsulate a high payload of therapeutic molecules that accumulate at the tumor site, both to enhance the local drug concentration and to reduce peripheral cytotoxic side effects^12^. However, although targeted drug delivery systems have been widely used for cancer therapy, these systems have only recently gained interest in promoting healing/regeneration of damaged cardiac tissue^13^.

In addition to drug delivery, targeting ligands have also been applied for visualization of specific cells by optical methods^14^, magnetic resonance imaging (MRI)^15^ or positron emission tomography (PET)^16^. Specific cell tracking by MRI has several advantages, because MRI is free of radiation and offers high spatial resolution and excellent contrast between soft tissues. However, single cells or clusters of cells within a tissue often do not show physical properties which can be exploited by MRI to generate a cell-specific contrast. For this reason, MRI contrast agents based on iron oxide or gadolinium have been utilized that alter the relaxation properties of endogenous ^1^H protons and therefore allow to visualize individual cell types^17^. Recently, fluorine 19 (^19^F) has emerged as new class of MR contrast agents. ^19^F has the second highest sensitivity of all MR-active nuclei and is nearly absent from biological tissue^18^. Consequently, accumulation of ^19^F atoms can be visualized with high sensitivity and specificity. Moreover, ^19^F MRI does not interfere with the signals of anatomical ^1^H MRI and therefore both datasets can be merged to gain information on the precise spatial localization of the ^19^F signal^19^. Perfluorocarbons are often used as ^19^F-source. These are fluorinated organic molecules that carry a high payload of ^19^F atoms. Perfluorocarbons are not soluble in water and instead are emulsified with lipids to form stable perfluorocarbon nanoemulsions (PFCs) with droplet sizes of 100–200 nm. Intravenous administration of PFCs leads to strong phagocytic uptake by monocytes and macrophages that accumulate at inflammatory lesions which has been successfully used for imaging inflammation in diseases like myocardial infarction, myocarditis, transplant rejection or pneumonia by combined ^1^H/^19^F MRI^20,21^. Interestingly, ^19^F MRI has also been used in theranostic applications since combining targeted drug delivery with imaging offers the unique possibility to determine if and how much of the therapeutic agent reaches the desired target tissue and how this impacts disease development^22^.

As described above, EpiSCs can differentiate into multiple cell types but their regenerative potential in particular in the adult heart of mammals is still unclear ^4,9,23,24^. Therefore, a system would be highly desirable that allows for targeted delivery of: (i) contrast agents to allow for noninvasive molecular imaging, and (ii) the delivery of small molecules to manipulate cell activity and/or fate. Since there is currently no specific ligand for EpiSCs known, the aim of the present study was to utilize a differential phage display screening approach to identify small peptides which specifically bind to EpiSCs but not to circulating immune cells and to exploit these peptides for the cell specific delivery of an ^19^F contrast agent to EpiSCs.

## Materials and methods

### Animal experiments and ethics

Animal experiments were performed in accordance with German Guidelines on animal care, and were approved by the Landesamt für Natur-, Umwelt-, und Verbraucherschutz (LANUV) (Reference number: 84–02.04.2014.A174). For this study, male Wistar rats (body weight, 220–280 g; age, 12–16 weeks) were housed at the central animal facility of the Heinrich-Heine-Universität Düsseldorf (ZETT, Düsseldorf, Germany), on a 12 h light/dark cycle and were fed with a standard chow diet and received tap water ad libitum.

Blood was withdrawn from healthy human volunteers by venous puncture to isolate primary monocytes. The study conformed to the Declaration of Helsinki and was approved by the University of Düsseldorf Ethics Committee (No. 2020–989). Informed consent was obtained from all volunteers.

### Isolation and cultivation of EpiSC

Myocardial infarction was induced by transient ligation of the left coronary descending artery. Rats were intubated and mechanically ventilated with 40% oxygen in air, anesthesia was induced with 3% (vol/vol) and maintained with 1.5% (vol/vol) isoflurane (Abbott, Chicago, USA). Ischemia was maintained for 60 min and the tissue was reperfused before the chest was closed^5^. EpiSCs were isolated 5 days after MI according to protocols described previously^5,25^. In brief, rats were euthanized in deep anesthesia induced with Ketamine (100 mg/kg BW; Ketaset, 100 mg/ml, Zoetis, Berlin, Germany) and Xylazine (10 mg/kg BW; Rompun 2% injection solution, Bayer AG, Leverkusen, Germany) at a total volume of 10 ml/kg BW. Hearts were excised and after being rinsed with saline, the surface of the hearts was digested with collagenase-containing solution (1 200 U/ml; CLS II, Biochrom GmbH, Berlin, Germany) under gentle rocking (20 rpm) at 37 °C for 20 min. The resulting cell suspension was collected and passed through a 100 µm cell strainer, centrifuged (350 × g, 5 min) and the cells were resuspended in culture medium [DMEM (high glucose; Sigma Aldrich Chemie GmbH, Munich, Germany) with 30% Fetal Bovine Serum (FBS; Biochrom GmbH), 1% sodium pyruvate (100 mM; Invitrogen GmbH, Meerbusch, Germany), 1% Penicillin–Streptomycin (10 000 U/ml; Biochrom GmbH), 1% Glutamax (100 x; Invitrogen GmbH), 1% HEPES-buffer (1 M; ThermoFisher Scientific, Meerbusch, Germany)]. Cells were plated in cell culture flasks (T175; Greiner Bio-One GmbH, Frickenhausen, Germany). After one day of culture in an incubator at 37 °C and 5% CO_2_ in a humidified atmosphere, cells were washed with phosphate-buffered saline (PBS; w/o Calcium and Magnesium; NeoFroxx, Einhausen, Germany) and fresh medium was added. For prolonged EpiSC cultivation, medium was replaced every 2 days and cells were split at a confluency of about 80%.

### Biopanning of a phage library on cultivated EpiSCs and whole blood

A PhD7 phage library (10^9^ different phage clones; New England Biolabs GmbH, Frankfurt am Main, Germany) was used for the biopanning procedure on EpiSCs which had been seeded 48 h before. 5 × 10^10^ plaque forming units (PFU) were incubated for 1.5 h at 37 °C in a volume of 1 ml cell culture medium on 3 × 10^5^ EpiSCs. During the incubation time, cells were gently agitated. After incubation, the supernatant was discarded, and cells were washed 20 times with 5 ml of PBS. Bound phage were eluted at low pH (0.2 M Glycine–HCl, 1 mg/ml BSA, PBS; pH 2.2) for 15 min at 20 °C. The elution was stopped by neutralization with Tris–HCl (0.5 M, pH 7.4; Sigma-Aldrich Chemie GmbH).

To select peptides that bound to EpiSCs but not to circulating immune cells, we performed a second biopanning step to remove the phage clones that might also bind to leukocytes. To this end, a second panning was performed on rat whole blood. Phage originally eluted from EpiSCs were incubated for 1 h at 37 °C in 1 ml of whole blood. Thereafter, the samples were centrifuged (2000 × g, 10 min, 4 °C) and the supernatant, containing only EpiSC-specific phage, was frozen until further use.

### Determination of the phage titer by qPCR

The phage titer in all samples was determined by qPCR. To this end, phage-specific primers, which cover the insertion sequence, were utilized: pIII No 3 (fwd)5′-cgc aat tcc ttt agt ggt acc ttt c-3′; pIII No 4 (rev) 5′-cca gac gtt agt aaa tga att ttc tgt-3′ (BioSpring). The phage library was used at defined concentrations of 10^9^—10^2^ PFU for standard curve generation. Primers were used in a concentration of 10 µM for the amplification.

For titer determination, 1 µl of the phage samples was subjected to qPCR with the use of a Quanti Fast SYBR Green PCR Kit (Quiagen, Hilden, Germany) in a StepOnePlus Real-Time PCR System (Applied Biosystems, Meerbusch, Germany) according to the following protocol: Initial denaturation 98 °C, 30 s.; Denaturation 98 °C, 10 s.; Annealing 50 °C, 30 s.; Elongation 72 °C, 15 s., Terminal Elongation 72 °C, 5 min ; 40 cycles.

### Deep sequencing

Phage clones isolated after the second round of biopanning  were precipitated over night at 4 °C with 2 mM NaCl-PEG-8000 (Sigma-Aldrich Chemie GmbH). Subsequently, PCR was performed using deep sequencing primers (see supplementary Table 1) and the quality of the PCR products was assessed using a fragment analyzer (AATI, Agilent Technologies Deutschland GmbH, Ratingen, Germany). Deep sequencing was performed with an Ion Torrent PGM (ThermoFisher Scientific) and a 318 v2 sequencing chip. For analysis of the phage sequences, TorrentSuite 5.2.2 software (ThermoFisher Scientific) was used and downstream bioinformatics sequence analyses were performed using PHASTpep^26^. This software uses a portion the sequence data that corresponds to displayed peptides and removes sequences that do not have the codons present in NEB libraries^26^. DNA sequences are translated into amino acid sequences and frequencies are calculated and normalized to the reference library frequencies for each peptide. The program sorts in such a way that sequences selective for a particular target rise to the top fraction.

### EpiSC specific peptides (EPs)

Five EpiSC-specific peptides (EPs) were synthesized commercially (Genaxxon) with a C-terminal GGGK(FAM)C. The fluorescent dye carboxyfluorescein (FAM) was coupled to the ε-amino group of the lysine to enable flow cytometry and fluorescence imaging and the terminal cysteine was used for conjugation to PFC.

### Binding of EPs to EpiSC and immune cells

#### Cultured EpiSC

EpiSCs were detached from the cell culture flask with PBS-EDTA (5 mM) and resuspended in DMEM at a concentration of 0.5 × 10^6^ cells/ml. Cell suspensions were incubated with 5 µg/ml of EP1, EP2, EP3, EP7 and EP9 for up to 120 min at 37 °C. After 10, 20, 40, 80 and 120 min, samples were transferred into tubes containing ice-cold MACS buffer (PBS, 2% FCS, 1 mM EDTA) to stop the reaction. Cells were centrifuged for 5 min at 500 × *g* and 4 °C, washed twice and finally suspended in MACS buffer with DAPI (4′,6-Diamidino-2-phenylindole dihydrochloride; 1 µg/ml) to label dead cells. Flow cytometric analysis was performed on a BD FACS Canto II (BD Biosciences, Heidelberg, Germany).

#### Rat immune cells

To investigate the binding of EPs to rat immune cells, five ml of rat blood was obtained by puncture of the caudal *vena cava* and subjected to erythrocyte lysis by hypotonic buffer (0.31 mM ammonium chloride, 10 nM potassium hydrogen carbonate, 0.201 mM Na-EDTA, add dist. water to 500 ml) for 10 min at 4 °C. Erythrocyte lysis was repeated for three times, cells were suspended in culture medium and then used for experiments. The labelling with EPs and subsequent analysis was carried out as described above for EpiSCs.

#### Freshly isolated EpiSC

As cultured EpiSCs might be different from freshly isolated EpiSCs, we also tested peptide binding on EpiSCs directly after preparation. The EpiSCs cell suspension obtained by the isolation procedure (see above) was incubated with EPs as described above. After incubation, the reaction was stopped by adding ice-cold MACS buffer. To distinguish EpiSCs and immune cells, the cell suspension was centrifuged for 5 min at 500 × g at 4 °C, washed with MACS buffer, suspended in CD45-PE (PE Mouse anti-rat CD45, Clone OX-1, BD Bioscience) and CD11b/c-PE/Cy7 (PE/Cy7 anti-rat CD11b/c, Clone OX-42, BioLegend) antibody solution and incubated for 30 min at 4 °C. To verify that the CD45/CD11b negative population represents EpiSCs, we also analyzed the CD73 expression (anti-CD73, Clone 5F/B9, BD Pharmingen and anti-goat Alexa488 secondary Ab, Life Technologies). Flow cytometric analysis was performed after two washing steps with MACS and staining with DAPI (1 µg/ml) on a BD FACS Canto II.

#### Human monocytes

Blood was withdrawn from healthy human volunteers by venous puncture and 10 ml of whole blood were mixed with 10 ml of PBS and gently laid on top of Histopaque-1077 (Sigma-Aldrich Chemie GmbH). After density centrifugation, the PBMC layer was diluted 1:1 with PBS and a centrifugation step was performed. The cell pellet was subjected to erythrocyte lysis using hypotonic buffer for 10 min at 4 °C. After centrifugation, the cells were resuspended in 8.5 ml DMEM. The cell suspension (600 µl) was incubated with 1.9 µg of EP9 for 45 min, at 37 °C. After 5, 15 and 45 min, samples were transferred into ice cold MACS buffer to stop the reaction and centrifuged for 5 min at 500 × g. Cells were washed with MACS buffer, resuspended in CD14-PE/Cy7 (PE/Cy7 anti-human CD14, Clone 63D3, BioLegend, San Diego, USA) antibody solution and incubated for 30 min at 4 °C. Flow cytometric analysis was performed after two washing steps and suspension in MACS buffer with DAPI (1 µg/ml) using a BD FACS Canto II.

### Binding studies of EPs on in vitro generated thrombi

Human whole blood was centrifuged for 5 min at 500 × g and the platelet-rich plasma (PRP) on top was carefully withdrawn. To induce thrombus formation, 1 ml of PRP was mixed with 100 µl CaCl_2_ (4 mM), 10 µl ADP (1 mM) and 100 µl thrombin (5 U/ml). To activate the PRP, the mixture (in 100 µl samples) was transferred to a 96-well plate and incubated for 1.5 h at 37 °C. Thrombi were detached from the wells with a tweezer and washed in 1 ml of PBS. For the incubation of thrombi with EPs, 500 µl PBS and 5 µg/ml peptide (EP1, EP2, EP3, EP7, EP9) were used per well of a 48-well plate. As positive control, a peptide was used which is based on the fibrin-binding conjugate EP2104R^27^ (here termed Fbn). Thrombi were incubated with peptides for 30 min at room temperature, washed three times with 500 µl of PBS and the fluorescence signal of the peptides bound to the thrombi was analyzed using an IVIS Lumina II Imaging System (Perkin Elmer, Rodgau, Germany).

### Interaction kinetics by surface plasmon resonance (SPR)

To analyse the interaction kinetics between peptide EP9 and EpiSCs, we used a BIAcore X100 system equipped with a CM5 sensor chips (GE Healthcare GmbH, Solingen, Germany). EP9 peptide was immobilized to the surface of the CM5 chip (600 µg/ml; 30 µl/min) using a thiol coupling chemistry (GE Healthcare GmbH). Presence of a free SH group at the C-terminal of EP9 peptide enabled us to efficiently immobilize it through a thiol coupling procedure. Afterward, EpiSCs and THP-1 monocytes (ATCC: TIB-202; 10,000 cells/µl) were injected over the sensor chip to monitor the association between immobilized EP9 peptide and the cells. After injection of the cells, dissociation phase was monitored by introduction of buffer for a period of 300 s. In another experimental strategy, increasing numbers of EpiSCs (200, 400 and 800 × 10^3^) were flushed over the immobilized peptide EP9 with a flow rate of 30 µl/min and dissociation was measured for an identical period of time. Finally, to block the EP9 binding sites, EpiSCs were pre-incubated for 30 min with 5 µg of EP9 before injection into the BIAcore system and subjected to the same procedure as described above. All SPR experiments were carried out at 25 °C in PBS.

### Preparation of perfluorocarbon nanoemulsions (PFC)

#### Fluorescent PFCs

We have prepared several kinds of PFCs which contained fluorescent lipids to enable flow cytometry to determine the cellular uptake. These lipids were either rhodamine B-labelled [Lissamine Rhodamine B 1,2-Dihexadecanoyl-sn-Glycero-3-Phosphoethanolamine, Triethylammonium Salt (rhodamine DHPE); Molecular probes, L1392] or labelled with Cy5 [1,2-dioleoyl-sn-glycero-3-phosphoethanolamine-N-(Cyanine 5); Avanti-Polar Lipids].

PFCs containing the fluorescent dye Cy5 (^Cy5^PFCs) or rhodamine (^Rho^PFCs) were prepared as described previously^28,29^. The PFC preparations contained 10 mM phosphate buffer (7 mM Na_2_HPO_4_, 3 mM NaH_2_PO_4,_ pH 7.4 isotonized with 2.5% [w/w] glycerol), 35 mM phospholipid (Lipoid S75; Lipoid GmbH, Ludwigshafen, Germany) and 0.01 mol% Cy5- or rhodamine-labelled lipids. A pre-emulsion of this suspension was generated for 30 min on a magnetic stirrer at 300 rpm. Afterwards, 20% (w/w) of perfluoro-15-crown-5 ether (ABCR, Karlsruhe, Germany) was added and a crude emulsion was formed by high shear mixing. PFCs were generated by high pressure homogenization at 1000 bar, using a LV1 microfluidizer (Microfluidics Corp., Westwood, USA) and heat-sterilized in glass vials under standard conditions (121 °C, 1 bar, 22 min) and stored at 4 °C under light protection.

#### Manufacturing of targeted PFCs

For the active targeting of PFCs, we also explored different kinds of PFC modifications. On the one hand, we utilized PFCs which were prepared with maleimide-PEG_2000_-DHPE and therefore contained maleimide residues on the PFCs surface (^Mal^PFCs;^28^). To avoid hydrolytic degradation of the maleimide group these were stored at − 80 °C until use. All of the maleimide-PFCs also contained rhodamine-labelled lipids and PEGylated lipids (see below). On the other hand, we used the sterol-based post-insertion technique (SPIT) to functionalize the cell-surface of preformed ^Cy5^PFCs with Cholesterol-PEG_2000_ or a conjugate of Cholesterol-PEG_2000_-maleimide and the EpiSC binding peptides (see below).

##### Maleimide-PFCs

Maleimid containing PFC (^Mal^PFCs) for coupling reactions were prepared as previously reported^28^. Phosphate buffer (7 mM Na_2_HPO_4_, 3 mM NaH_2_PO_4,_ pH 7.4 isotonized with 2.5% [w/w] glycerol) and 35 mM phospholipid (Lipoid S75; Lipoid GmbH, Ludwigshafen, Germany) was mixed with 0.01 mol% rhodamine-labelled lipids (Rhodamine-DHPE; Molecular Probes, ThermoFisher Scientific), 4.5 mol% PEGylated lipids (DSPE-PEG_2000_; Lipoid GmbH) and 0.05 mol% maleimide lipids (DSPE-PEG_maleimide_; Avanti Polar Lipids Inc., Alabaster, USA). The nanoemulsions were then generated by high pressure homogenization as described above.

^EP9^PFCs were prepared using ^Mal^PFCs in a one-step reaction. To this end, 100 µl of ^Mal^PFCs were thawed on ice, mixed with 100 µl phosphate buffer and incubated over-night with 25 µg of EP9.

##### Sterol-based post insertion

^Cy5^PFCs were either (i) PEGylated or (ii) PEGylated and tagged with EP9 by sterol-based post-insertion which is based on the spontaneous insertion of cholesterol-conjugates into the lipid layer of liposomes or PFCs^30,31^. First, the optimal amount of PEGylation was tested using different amounts of cholesterol-PEG_2000_-CH_3_. Therefore, 100 µl of ^Cy5^PFCs were incubated for 3 h on a shaker at room temperature with 20, 40 or 80 µl of cholesterol-PEG_2000_-CH_3_ (2 µg/µl; Nanocs Inc.). Phosphate buffer was added until a total volume of 200 µl was reached.

To prepare ^EP9^PFCs by SPIT, we first incubated 15 µl of EP9 (5 µg/µl) with 40 µl cholesterol-PEG_2000_-maleimide (1 µg/µl) and 5 µl phosphate buffer for 1 h on a shaker at room temperature. We then added 40 µl cholesterol-PEG_2000_-CH_3_ (3 µg/µl). This suspension was incubated on a shaker for 1 h at room temperature. Finally, 100 µl of undiluted Cy5-PFCs were added and the mixture was incubated for 3 h at room temperature to enable the spontaneous insertion of the cholesterol-EP conjugate into the PFC lipid layer.

### Analysis of the conjugation specificity of ^Mal^PFCs

For preparation of ^EP9^PFCs, 100 µl of ^Mal^PFCs and 100 µl of phosphate buffer were incubated over-night with increasing amounts of EP9 (12.5 µg, 25 µg or 50 µg). Purification of the ^EP9^PFCs was performed by gradient centrifugation with Histopaque-1119 (Sigma-Aldrich Chemie GmbH). During centrifugation, PFC droplets accumulate at the bottom of the tube because of the high density of the perfluorocarbons (up to 2 mg/ml), whereas soluble ligands remain in the aqueous phase above the ficoll layer. After density centrifugation, the supernatant was removed and the purified PFCs were resuspended in PBS. As control, ^Rho^PFCs were also incubated with increasing amounts of EP9 and purified by ficoll gradient. The fluorescence signals of the ^Rho^PFCs and ^Mal^PFCs with EP9 was analyzed by IVIS Lumina II (PerkinElmer).

### Cellular uptake of ^EP^PFCs

#### Cellular uptake of ^EP9^PFC by EpiSC and THP-1 monocytes

EpiSCs were detached from the cell culture flask using PBS-EDTA (5 mM) for 15 min at 37 °C and finally resuspended in EpiSC culture medium at a density of 1 × 10^6^ cells per ml. EpiSCs were centrifuged, resuspended in 600 µl of cold MACS buffer and 5 µl of ^EP9^PFCs (rhodamine-labelled) were added. The suspension was incubated for up to 120 min at 37 °C. After 10, 20, 40, 80 and 120 min, samples were transferred into tubes containing ice-cold MACS buffer to stop the reaction. Cells were centrifuged for 5 min at 500 × g at 4 °C, and flow cytometric analysis was performed after two washing steps and resuspension in MACS buffer with DAPI (1 µg/ml) to label dead cells. Flow cytometric analysis was performed on a BD LSR Fortessa (BD Biosciences). To further characterize the specificity of the ^EP9^PFC, EpiSCs and THP-1 monocytes (10^6^ cells of each) were mixed, 5 µl of ^EP9^PFCs were added and the cells were incubated for 30 min at 37 °C. After incubation, 2 ml of ice-cold MACS buffer was added to the suspensions and the samples were centrifuged for 5 min at 500 × *g* and 4 °C. The supernatant was discarded, the samples were washed twice with 200 µl of MACS buffer and stained with DAPI (1 µg/ml) to label dead cells. Flow cytometry was performed on a BD LSR Fortessa.

#### Cellular uptake of ^EP9^PFCs by EpiSCs and primary human monocytes

To compare the cellular uptake of ^Cy5^PFCs and PEGylated ^Cy5^PFCs as well as Cy5-labelled ^EP9^PFCs by human monocytes and EpiSCs, cell suspensions (600 µl with 10^6^ cultured EpiSCs or human PBMCs) were incubated with 5 µl Cy5-PFCs, ^PEG^Cy5-PFCs, or ^EP9^Cy5-PFCs for up to 45 min at 37 °C. After 5, 15 and 45 min, samples were transferred into tubes containing ice cold MACS buffer to stop the reaction and centrifuged for 5 min at 500 × g. To identify monocytes, PBMCs were stained with CD14-PE/Cy7 mAb (1 µg/ml; PE/Cy7 anti-human CD14, Clone 63D3, BioLegend). Flow cytometry analysis was performed after two washing steps with MACS buffer and cells were suspended in MACS buffer with DAPI (1 µg/ml) and analyzed on a BD FACS Canto II.

#### ^19^F magnetic resonance imaging (^19^F MRI)

EpiSCs were seeded on a 10 cm culture dish and cultivated until the cells achieved a confluency of 90%. PFCs without targeting ligand (^Mal^PFCs) or ^EP9^PFCs based on ^Mal^PFCs (see above) (50 µl) were added to fresh medium (10 ml) on top of the cells and incubated over-night at 37 °C in a humidified CO_2_ incubator. On the next day, cells were detached with PBS-EDTA (5 mM) and fixed with PFA (4%) for 10 min on ice. After centrifugation for 5 min at 500 × g, 200 µl of PBS were carefully pipetted on the top of the cell pellet. The tube was subjected to ^1^H/^19^F MRI on a Bruker 9.4 T AVANCEIII wide bore NMR spectrometer (Bruker BioSpin MRI GmbH, Ettlingen, Germany). The cell pellet was visualized by T2 weighted ^1^H MRI [micRARE; FOV = 2.56 cm^2^, Thickness = 1 mm, Matrix: 128 × 128, RARE factor 16, TR (repetition time) = 3500 ms]. Corresponding ^19^F MRI was conducted using a standard ^19^F RARE sequence with the following parameter: FOV = 2.56 cm^2^, Thickness = 3 mm, Matrix = 32 × 32, TR = 2500 ms; Averages = 3000.

### Characterization of physicochemical properties, stability and cytotoxicity

#### Physicochemical properties of PFCs, ^PEG^PFCs and ^EP9^PFCs

PFCs, ^PEG^PFCs and ^EP9^PFCs were generated as described above (see 2.10). Subsequently, 100 µl of PFCs, ^PEG^PFCs or ^EP9^PFCs were mixed with 300 µl of phosphate buffer or cell culture medium. 50 µl of the PFCs were diluted with 500 µl of milliQ water and analyzed by dynamic light scattering (DLS; Nanotrac Wave II, Microtrac MRB) to determine the hydrodynamic diameter, the polydispersity index (PDI) and the ζ-potential.

#### In vitro* stability of *^*EP9*^*PFCs*

^EP9^PFCs were prepared above and mixed with phosphate buffer or cell culture medium and incubated at 4 °C or 37 °C for 6 h, 24 h and 48 h and analyzed by DLS.

#### Cyotoxicity

EpiSC and THP-1 cells were incubated with EP9 peptide or ^EP9^PFCs (200 µl of cells + 5 µl ^EP9^PFCs or 1.9 µg EP9) over a period of 120 min at 37 °C. Samples were withdrawn after 0 min, 15 min, 30 min, 60 min and 120 min and analyzed by flow cytometry. Cells were stained with DAPI (1 µg/ml) to label dead cells.

### Impact of storage on EP9/^EP9^PFC targeting efficacy

EP9 and ^EP9^PFCs were stored in phosphate buffer at 4 °C for 6 h and 24 h. Subsequently EP9 and ^EP9^PFCs were incubated on cultured EpiSCs for 30 min at 37 °C. Cells were washed twice with MACS buffer and then analyzed by flow cytometry.

### Data analysis and statistics

Analysis of qPCR data was performed with StepOne Plus software (ThermoFisher Scientific). Flow cytometric analysis was done with FlowJo 7.6.1 (BD Biosciences) and surface plasmon resonance data were evaluated using BIAevaluation (version 2.0.1; GE Healthcare GmbH). For analysis of the phage sequences, TorrentSuite 5.2.2 software (ThermoFisher Scientific) was used and downstream bioinformatics analyses were performed with PHASTpep^26^.

Data are presented as means ± SD. Student’s t test or two-way ANOVA were used to determine statistical significance. Differences were considered to be statistically significant at *p* ≤ 0.05 (* ≤ 0.05; ** ≤ 0.01; *** ≤ 0.001). The Prism software package (Version 7.0; GraphPad Software, San Diego, USA) was used for the statistical analysis.

## Results

### Identification of EpiSC-specific phage by differential biopanning and next generation sequencing

**Figure 1:**
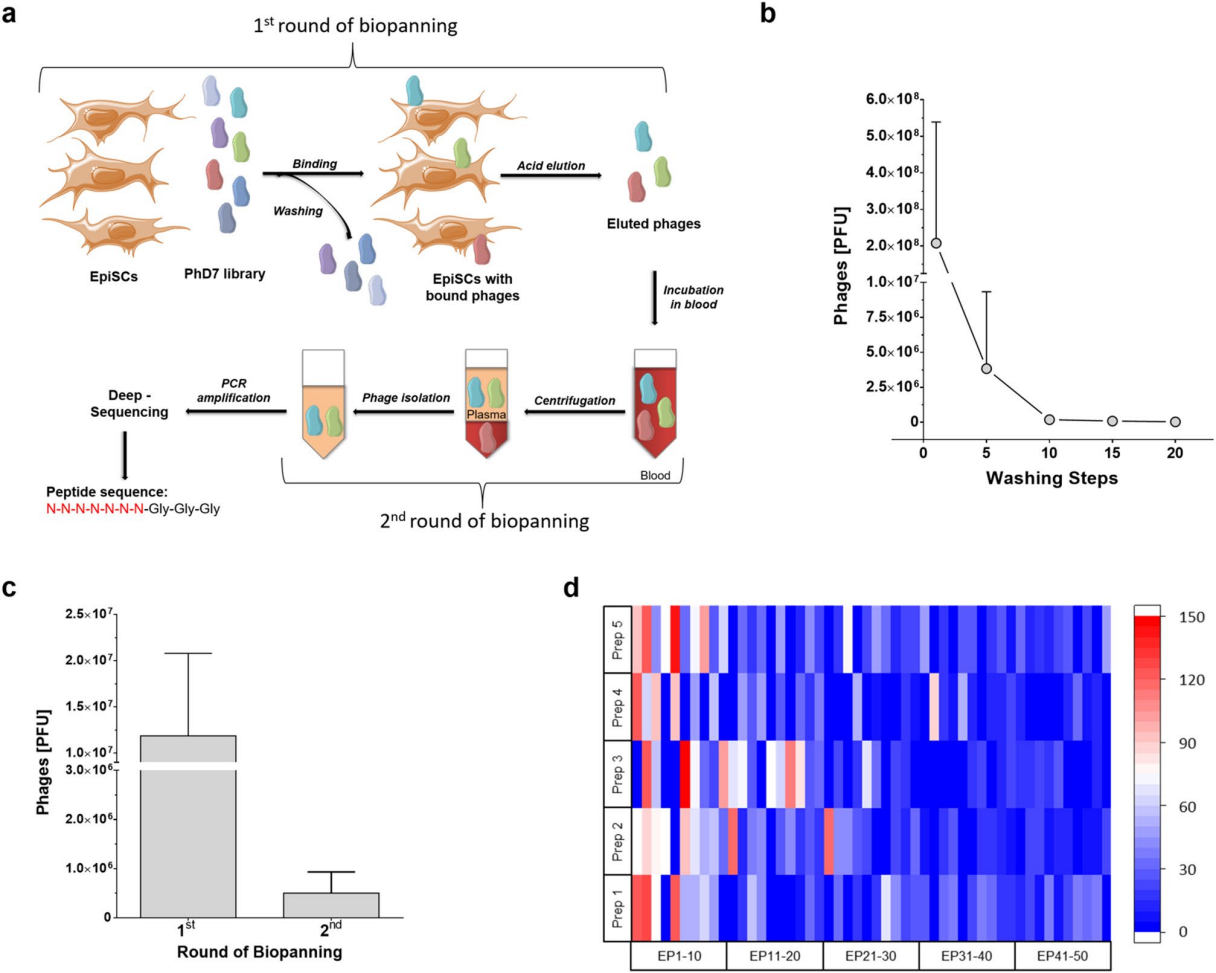
(**a**) Schematic overview of the phage-display screening approach: phage from the PhD7 library were incubated with cultured EpiSCs, intensively washed and eluted at low pH (1st round of biopanning). Subsequently, isolated phage were incubated with blood to remove phage clones that bound to cellular components of blood (2nd round of biopanning). Phage isolated from the blood plasma were subjected to deep sequencing and bioinformatics analysis. (**b**) After incubation with the PhD7 phage library, EpiSCs were intensively washed and the amount of released phage per washing step was determined by qPCR (Mean ± SD; n = 5). (**c**) Total number of phage (± SD of n = 5) which were obtained after the first and second round of the biopanning procedure. (**d**) Enrichment of EpiSC specific peptides sequences (EP): the relative increase of the 50 most abundant peptide sequences in the five separate phage isolations from the 2nd round of the biopanning procedure. Enrichment was calculated in relation to the parent PhD7 phage library and is displayed as color-coded heat map. EPs (EP1-EP50) were arranged according to their mean relative enrichment. Prep = individual preparation number of EpiSCs. Parts of the figure were drawn by using pictures from Servier Medical Art (http://smart.servier.com/).

To identify peptides, which specifically bound to EpiSCs formed after MI, we utilized a two-step biopanning procedure as outlined in Fig. 1a. In the first step, a phage library was incubated with EpiSCs and bound phages were eluted. In the second step, eluted phages were incubated with rat whole blood to filter out those peptides which bound to blood components. After PCR amplification, the DNA fragments which correspond to the genomic region that encodes for the peptide sequences were analyzed by next generation sequencing (NGS) followed by bioinformatics analysis.

EpiSCs from five separate isolates—obtained from infarcted rat hearts 5 days post MI—were incubated for 1.5 h at 37 °C with 5 × 10^10^ PFU of a commercial PhD7 library. This corresponds to an average of 50 copies of each unique peptide sequence of the linear random 7-mer library. Next, the cells were washed 20 times with PBS and the number of the released phage was quantified by qPCR. As shown in Fig. 1b, a majority of low affinity binding phage were released within the first 10 washing steps. Subsequently, phage clones with high affinity to EpiSCs were detached at low pH (pH 2.2) followed by qPCR for phage titer determination (Fig. 1c). This first round of biopanning resulted in the recovery of about 10 × 10^7^ phages (11.9 × 10^7^ ± 8.9 × 10^7^; n = 5) which avidly bound to EpiSCs (positive selection). To enhance specificity, phage with a high affinity for EpiSCs were subjected to a second round of biopanning to eliminate those phage that may also bind to cellular components of the blood (negative selection). For the second round of biopanning 1 ml of rat whole blood was incubated with the phagepool for 1 h at 37 °C. Thereafter, blood plasma was separated from cellular blood components by centrifugation, and about 500 000 phage clones (5.0 × 10^6^ ± 4.3 × 10^6^, n = 5) could be recovered from the plasma fraction (Fig. 1c). This corresponds to 5.8% ± 5.6% of the clones that were obtained in the first biopanning step.

To determine peptide sequence identity of enriched phage, NGS using an Illumina platform was performed. We utilized barcode-sequence-labelled specific primer-pairs that cover the peptide insertion region of the pIII-sequence (see supplementary Table 1) for simultaneous sequencing of five separate phage pools isolated from the second round of biopanning, and the parent PhD7 library as control. For quality control of the PCR reaction, we performed fragment analysis, which revealed highly specific products with only a single PCR fragment of the expected length (Fig. S1). NGS identified a total of 4.8 × 10^7^ insertion sequences and PHASTpep^26^ was used to extract enriched peptide sequences from the dataset. A total of 0.3–1 × 10^6^ individual peptide sequences were identified and enrichment was calculated as fold increase compared to the parent PhD7 library (Fig. 1d). The relative abundance of the 50 most prevalent peptide sequences from each of the five EpiSC isolates is displayed as color-coded heatmap in Fig. 1d (a list of the fifty most abundant sequences is provided as supplemental Table 2).

### Identified peptides bind to EpiSCs, but not to immune cells

On the basis of the identified EpiSC-specific peptide sequences, we selected five lead peptides, which were found in all EpiSC samples and displayed a mean relative abundance of more than a 40-fold enrichment compared to the parent library. Peptide sequence and the relative enrichment of EP1, EP2, EP3, EP7 and EP9 are listed in Table 1.

**Table 1:** EpiSC-specific peptides (EP): lead peptides selected from the pool of peptide sequences obtained from the second round of the biopanning procedure. Displayed is the number of the peptide (EP), the peptide sequence and the mean relative enrichment (± SD) compared to the parent phage library.

Peptide	Peptide sequence	Rel. enrichment (Mean ± SD)
EP1	SEPIVPL	105.5 ± 61.9
EP2	ATKTIAP	104.9 ± 25.0
EP3	THVYRDE	84.5 ± 37.5
EP7	QSHALMA	63.4 ± 10.1
EP9	KLMLPRP	42.4 ± 14.0

**Figure 2:**
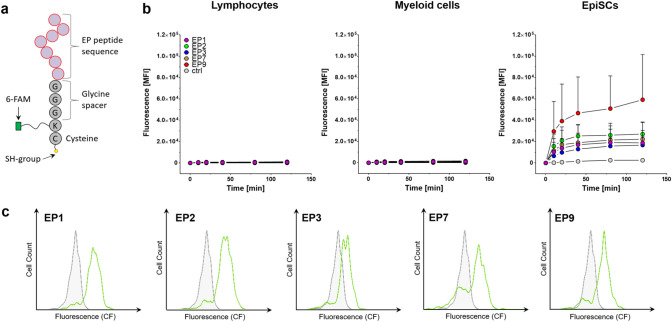
(**a**) Scheme of synthesized EP peptides. The 7-mer peptide sequence is followed by a spacer (GGGK) and a terminal cysteine with a free sulfhydryl group. Carboxyfluorescein (6-FAM) is linked to the ε-amino group of the lysine within the GGGK-spacer, to enable flow cytometry and fluorescence imaging. (**b**) Flow cytometric analysis of the time-course of binding of EPs to cultured EpiSCs (right) and blood-derived myeloid- (middle) and lymphoid (left) immune cells. Data are shown as mean fluorescence intensity ± SD; n = 5. (**c**) EP-binding to freshly isolated EpiSCs. The cell suspension was stained with mAbs against CD45, CD11b and CD73 to identify EpiSCs (CD45− , CD11b−, CD73+ and incubated with either EP1, EP2, EP3, EP7, EP9 or left untreated as control. The histogram plots show an overlay of EP-treated cells (green) and untreated controls (grey). Representative histogram plots of n = 3 experiments.

EP1, EP2, EP3, EP7 and EP9 peptides were synthesized commercially and modified with carboxyfluorescein (6-FAM) at the ε-amino group of the lysine in the GGGK-spacer to enable flow cytometry and microscopy. A cysteine was added to the C-terminus of the GGGK-sequence to enable conjugation to nanoemulsions (Fig. 2a). In a first set of experiments, we tested the binding of these modified EPs to cultured EpiSCs as well as to blood-derived rat immune cells by flow cytometry. As shown in Fig. 2b, all of the EPs bound to EpiSCs but EP9 displayed the best binding properties. Importantly, we did not observe any binding of EPs to myeloid cells (CD45+/CD11b+) or lymphocytes (CD45+/CD11b−). Statistical analysis revealed that EP9 shows superior binding to EpiSC compared to EP1,-2,-3 and EP7 (see Table S3).

EPs were initially selected by their binding potential to cultivated EpiSCs. To more closely mimic the in vivo situation, we also investigated the binding properties of EPs to freshly isolated and noncultured EpiSCs. Cells were obtained by collagenase digestion of the surface of infarcted rat hearts (for details see Methods) and this cell suspension was incubated with EPs for 30 min. EpiSCs were identified by flow cytometry based on FSC/SSC properties, the lack of CD45- and CD11b-expression and high levels of CD73 as previously described^5^. We observed that all EPs also bound to freshly isolated EpiSCs (Fig. 2c). Additionally, we tested peptide binding to platelets and activated platelets/thrombi. Platelets are present in the circulation in large numbers, accumulate in infarcted hearts and therefore must be ruled out as potential nonspecific target of the EPs. To this end, we incubated EPs or the fibrin-binding peptide (fbn) as control with in vitro generated white thrombi. Whereas the fibrin-specific peptides produced strong fluorescence signals in the thrombi, only low binding for EPs was observed (Fig. S2).

### Surface plasmon resonance analysis of the EP9/EpiSC interaction

**Figure 3:**
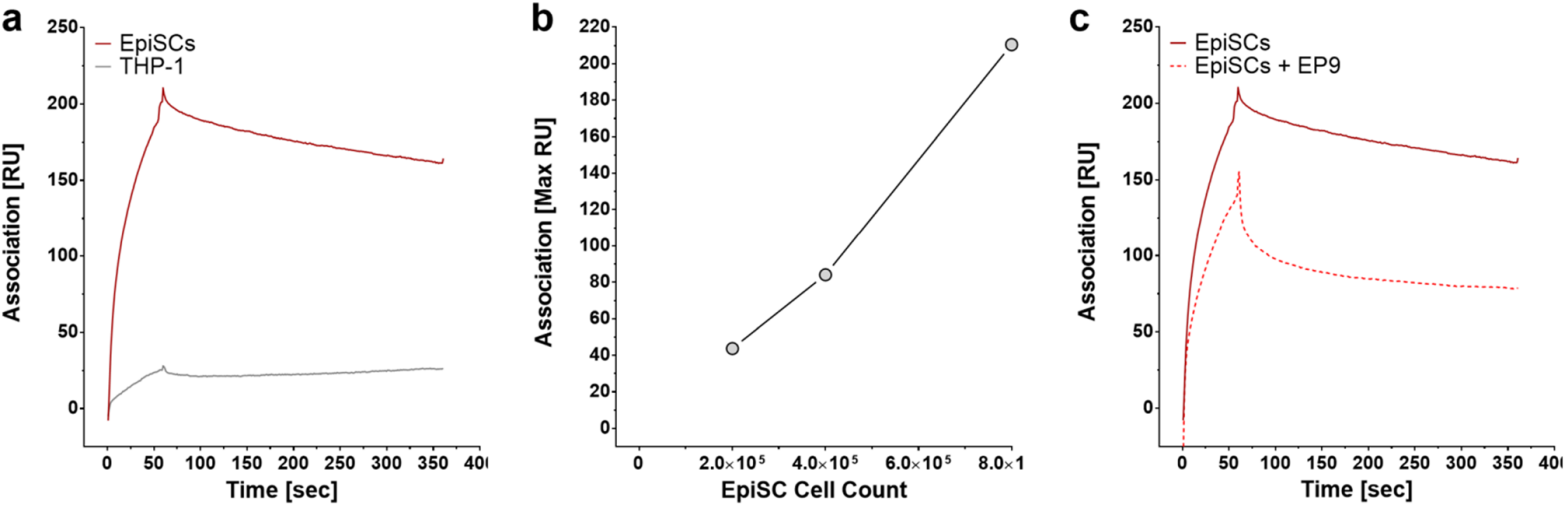
Surface plasmon resonance (SPR) of the EP9/EpiSC interaction: (**a**) EP9 was coupled to a BIAcore CM5 chip via the thiol coupling of C-terminal cysteine. EpiSCs, were flushed over the immobilized peptide EP9 and the binding was observed over a period of 60 s (Response Units = RU). Dissociation was induced after 60 s. by injection of buffer over the sensor chip and then dissociation was monitored over the following five min. As control, binding and dissociation of THP-1 monocytes (black curve) to immobilized EP9 was analyzed. (**b**) Impact of cell number on binding to EP9. Three different amounts of EpiSCs (200 × 10^3^, 400 × 10^3^, 800 × 10^3^) were assessed. The relation of the maximum response unit versus cell concentration is shown. (**c**) Competition assay: overlay of the SPR sensorgrams for EpiSCs (red) and EpiSCs pretreated with EP9 (red-dashed) to block the interaction with the BIAcore-EP9 chip.

To explore the kinetics behavior of EP9 binding in more detail, surface plasmon resonance (SPR) was applied. To this end, EP9 was covalently coupled to the surface of a CM5 chip via the C-terminal cysteine. Next, EpiSCs or a monocytic control cell line (THP-1) were passed over the sensor chip at a constant flow rate of 30 µl/min. As shown in Fig. 3a, binding curves of EpiSCs reveal that the cells rapidly associated to the surface thiol-immobilized peptide EP9 and exhibited a slow dissociation rate (0.005 s^−1^). In contrast, THP-1 monocytes showed only minor association to immobilized EP9. The observed binding of EpiSCs to EP9 was dependent on the cell number injected into the system (Fig. 3b). Binding could be detected for as little as 2 × 10^5^ EpiSCs. To further explore the specificity of the EP9/EpiSC interaction, we performed a competition assay (Fig. 3c). To this end, EpiSCs were pre-incubated with EP9 (5 µg/ml) for 30 min and subsequently subjected to SPR. Pre-incubated EpiSCs showed decreased rates of association and dissociation (0.024 s^−1^).

### ^EP9^PFCs mediated delivery of ^19^F contrast agent to EpiSCs

**Figure 4:**
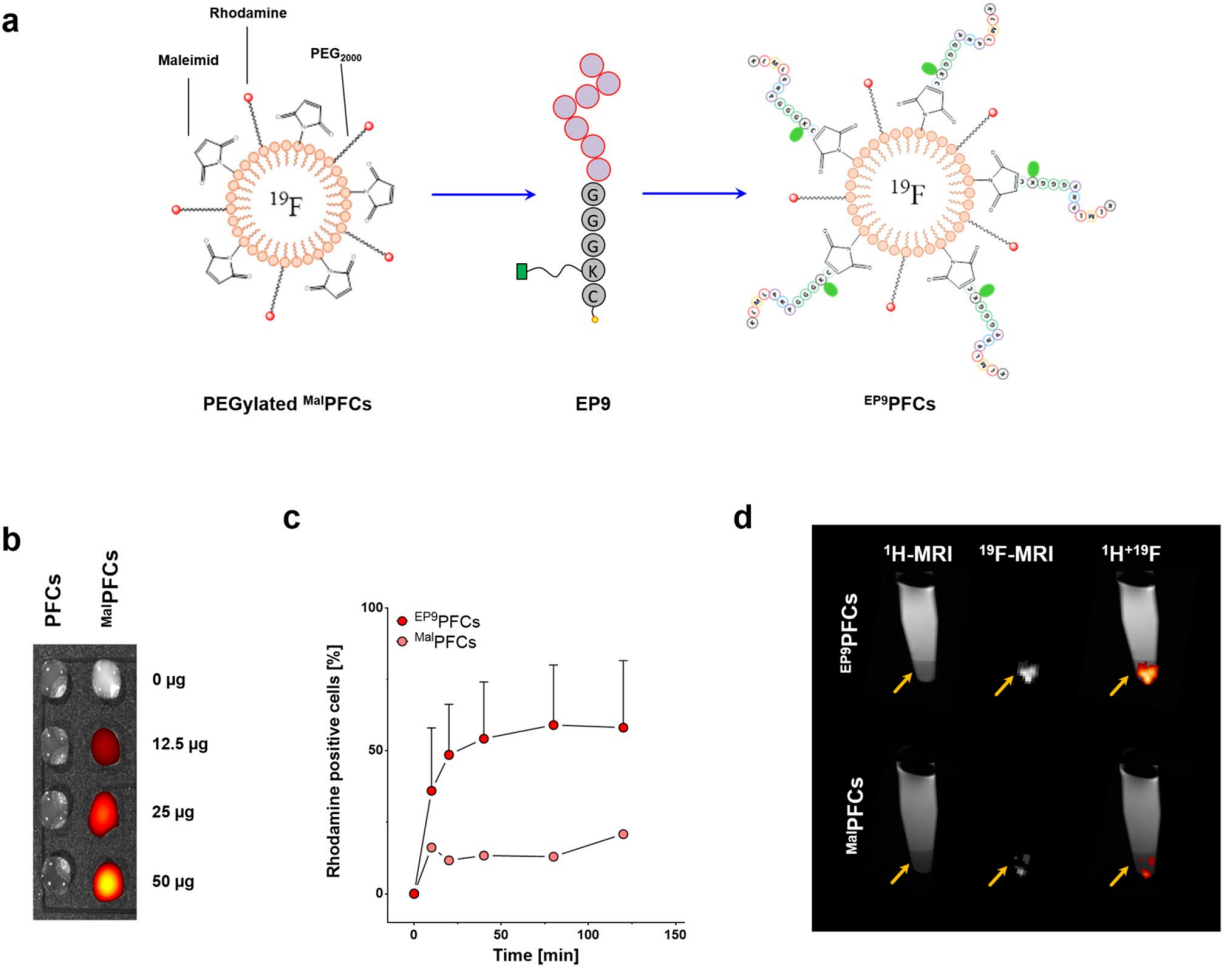
(**a**) Coupling of EP9 to maleimide-PFCs (^Mal^PFCs): ^Mal^PFCs are pre-formed PFCs composed of a single lipid layer, which encapsulates liquid perfluoro-15-crown-5 ether. The surface layer contains PEG_2000_-maleimide-lipids for coupling to sulfhydryl groups and rhodamine-labelled lipids to allow for fluorescence-imaging. (**b**) Increasing amounts of EP9 were incubated with ^Mal^PFCs or conventional ^Rho^PFCs to investigate the coupling of the free C-terminal –SH residues to the maleimide on the surface of ^Mal^PFCs. PFCs were purified by ficoll density gradient separation and the rhodamine fluorescence signal was analysed by IVIS. The image shows an overlay of the photograph (grey) with the carboxyfluorescein fluorescence image intensity (red to yellow). (**c**) Flow cytometric analysis of EpiSCs after incubation with ^EP9^PFCs (red) and nonspecific ^Mal^PFCs (light red) over a period of 120 min at 37 °C. Data show the relative amount of rhodamine positive cells (%) ± SD of n = 2–3 individual experiments. (**d**) Visualization of EpiSCs by ^19^F MRI: EpiSCs were incubated with ^EP9^PFCs (upper panel) or ^Mal^PFCs as control (lower panel), intensively washed with PBS, transferred to a reaction tube, pelleted by centrifugation and analyzed by combined ^1^H/^19^F MRI using standard ^1^H/^19^F RARE sequences. The left figure displays the anatomical ^1^H MRI scans (left), showing the pelleted EpiSC (bottom, arrow) which appear darker in T2-weighted MRI covered by the bright PBS (bright). In the middle figure the ^19^F scan is shown, and on the right the ^1^H and the ^19^F images have been superimposed.

Next, we sought to determine if EP9 conjugated to a PFC emulsion was capable of targeting ^19^F perfluorocarbons to EpiSCs. As a first step, we investigated whether attachment of EP9 to functional groups impairs the peptide’s binding properties. To this end, we coupled EP9 (which contains the fluorescent marker 6-FAM) to maleimide-PFCs (^Mal^PFCs) in a one-step reaction^28^. The SH-group of the C-terminal cysteine of EP9 reacts with a maleimide-residue on the PFC-surface to form a stable thioether (Fig. 4a). We verified the specificity of the conjugation by adding increasing amounts of the peptide to either ^Mal^PFCs and or to conventional ^Rho^PFCs, which do not possess maleimide residues on the surface. After incubation, the PFCs were purified by ficoll density gradient centrifugation, and the fluorescence signal of the peptide conjugated to PFCs was detected with IVIS (Fig. 4b). Increasing the peptide concentration resulted in stronger fluorescence signals for ^Mal^PFCs (Fig. 4b, right) but not for ^Rho^PFCs. This indicates that the EPs bind to ^Mal^PFCs by forming a thioether between the maleimide- and the sulfhydryl groups and do not adhere nonspecifically to the PFC surface (Fig. 4b, left).

To test binding of EP9-labelled PFCs (^EP9^PFCs) to EpiSCs, cells were incubated with ^EP9^PFCs and ^Mal^PFCs without peptide. A rapid and strong binding of ^EP9^PFC to EpiSC was observed, whereas ^Mal^PFCs showed only minor association (Fig. 4c). We also investigated if the ^19^F contrast agent is targeted to the EpiSCs in sufficient quantities to enable the visualization by ^19^F MRI. EpiSCs treated with ^EP9^PFCs and ^Mal^PFCs, were intensively washed and thereafter subjected to ^1^H/^19^F MRI. Figure 4d shows ^1^H MRI images of reaction tubes, containing ^EP9^PFC- or ^Mal^PFC-treated EpiSCs that were pelleted by centrifugation. The location of the cells on the bottom of the tubes is visible in T2-weighted MRI as a dark pellet that is covered by the bright signal of the phosphate buffered saline. The corresponding ^19^F image shows a very bright spot at the bottom of the ^EP9^PFC tube, whereas the ^19^F signal for the cells incubated with ^Mal^PFCs negative control is rather dim.

### Active targeting of EpiSCs outcompetes PFC-uptake by monocytes

PFCs are known to be preferentially phagocytized by monocytes and macrophages^19,20^. Therefore, we studied whether PFCs functionalized with EP9 are still taken up by monocytes, which might compete with labelling of EpiSCs in vivo.

**Figure 5:**
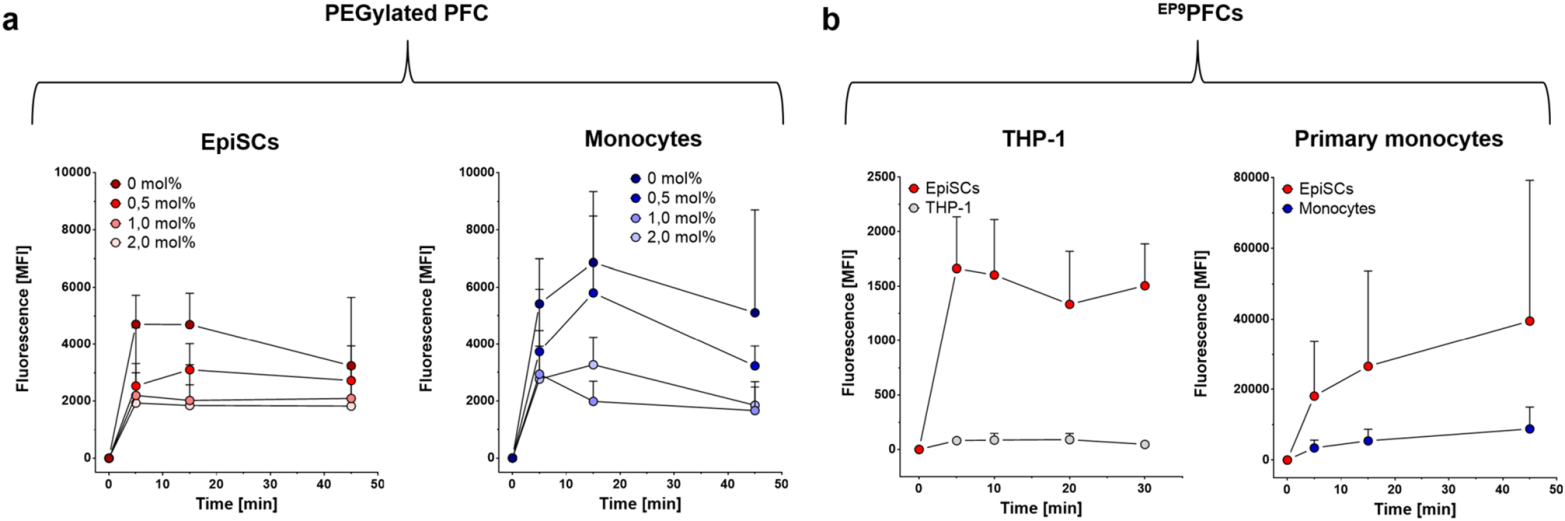
(**a**) Cellular uptake of PFCs with increasing amounts of PEGylation by EpiSCs and human primary monocytes. Cultivated EpiSCs and human primary CD14^+^ monocytes were incubated with ^Cy5^PFCs (0 µg cholesterol-PEG_2000_ = chol-PEG) and ^Cy5^PFCs which were modified with 0.5 mol%, 1 mol% and 2 mol% chol-PEG (referred to the total lipid content) over a period of 45 min at 37 °C. At distinct time points (0, 5, 15 and 45 min), the cells were analyzed by flow cytometry. Data show the Cy5 mean fluorescence intensity (MFI) of the cells ± SD of n = 3 experiments. (**b**) Competition of EpiSCs and THP-1 (left) or primary monocytes (right) for ^EP9^PFC uptake: Left: EpiSCs and THP-1 monocytes were incubated with ^EP9^PFCs (based on ^Mal^PFCs) over a period of 30 min at 37 °C. At distinct time points (0, 5, 10, 20 and 30 min), samples were analyzed by flow cytometry. Right: Cellular uptake of ^EP9^PFCs (generated by sterol-based post-insertion) by EpiSCs and human primary monocytes. Cells were incubated with ^EP9^PFCs over a period of 45 min at 37 °C and analysed after 0, 5, 15 and 45 min by flow cytometry. Data are shown as mean fluorescence intensity (MFI) ± SD of n = 3–5.

Reduction of the cellular uptake of conventional PFCs by phagocytic immune cells is important to enhance the specificity of targeting PFCs to other cell-types^32^. One of the most widely used agents to impair the cellular uptake of nanoparticles is the surface-modification with polyethylene glycol (PEG). PEGylation of nanoparticles generates a protective hydrophilic layer that repels the adsorption of opsonin proteins and therefore blocks or delays the opsonization process^33^. To impair the cellular uptake of PFCs by phagocytic cells we first analyzed the impact of PEGylation on the cellular uptake of Cy5-labelled PFCs. Therefore, we modified PFCs with increasing amounts of cholesterol-PEG_2000_ (chol-PEG; 0–2 mol% chol-PEG of the total lipid content) and tested the cellular uptake by EpiSCs and human blood monocytes. We found that the addition of 1–2 mol% chol-PEG led to an approximate 70% decrease in cellular uptake by EpiSCs (Fig. 5a, left) and monocytes (Fig. 5a, right). Interestingly, we observed that the fluorescence signal of ^Cy5^PFCs taken up by monocytes and EpiSCs slightly decreased over time most likely by a reduction of the fluorescence signal upon internalization and quenching in the late endosomal system^29^.

Next, we sought to determine if adding EP9 as a ligand to PEGylated PFCs was able to restore the labelling of EpiSCs yet avoid cellular uptake by monocytes. Therefore, we prepared EP9 targeted PFCs (^EP9^PFCs) which contained 1.5 mol% chol-PEG and 0.5 mol% EP9 coupled to cholesterol-PEG-maleimide. First, we performed a competition assay of EpiSCs with the human monocytic cell line THP-1 which revealed that ^EP9^PFCs are strongly taken up by EpiSCs but not by THP-1 cells (Fig. 5b, left). The mean fluorescence values of ^EP9^PFC-labelled EpiSCs after 30 min of incubation were 30-fold higher than of THP-1 monocytes (MFI 1507 ± 380 for EpiSCs and 46 ± 35 for THP-1 cells) (Fig. 5b). Since cell lines display weaker phagocytic properties, we also compared the cellular uptake by primary human blood monocytes and EpiSCs. Again, we observed a very rapid and strong uptake by EpiSCs (Fig. 5b, right) whereas the labelling of monocytes was much weaker, indicating an efficient retargeting of ^EP9^PFCs to EpiSCs. This is confirmed by the strong binding of the EP9-peptide to human EpiSCs whereas binding of EP9 to primary monocytes was not detectable (Fig. S3).

To further verify that the modification of PFCs with polyethylene glycol and the EP9-peptide are responsible for the observed effects, we analyzed the hydrodynamic diameter, the size distribution and the ζ-potential by dynamic light scattering (Supplemental Figure S4). The diameter of all PFCs is around 200 nm and the polydispersity index is 0.1 which indicates monodisperse distribution. Finally, the ζ-potential was − 30 mV with a slightly less negative potential for ^EP9^PFCs and PEGylated PFCs compared to unmodified PFCs.

For any targeting approach it is of crucial importance that the targeting probe is stable and does not display any cellular cytotoxicity. To this end, we analysed the physicochemical properties of ^EP9^PFCs over time, tested for cytotoxic effects of EP9 and ^EP9^PFCs and also analysed the impact of storage on the targeting efficacy. We found that size, size distribution and also the ζ-potential is stable at 4 °C and 37 °C over a period of 48 h even in the presence of serum (supplemental figure S5). Furthermore, we did not find evidence for acute cytotoxic effects of EP9 or ^EP9^PFCs (supplemental figure S6), but we observed that storage of ^EP9^PFCs or EP9 for 24 h severely reduced the cellular labelling of EpiSCs (Supp Figure S7).

## Discussion

In the present study, we aimed to develop a peptide-based system for the specific targeting of a ^19^F-based contrast agent to EpiSCs. Utilizing a differential phage display screen with 7-mer-peptides, we identified peptides that avidly bound to EpiSCs but not to circulating immune cells (EPs). Among the EPs so identified, EP9 in particular showed strong and rapid binding to cultured EpiSCs. In addition, coupling of EP9 to PFCs enabled the targeting of a ^19^F contrast agent to EpiSCs for visualization by ^1^H/^19^F MRI. Importantly, surface modification of PFCs with EP9 and PEG led to a strong cellular uptake by EpiSCs which out-competed PFC uptake by highly phagocytic human monocytes.

Antibodies are widely used for cell targeting because of their high specificity and affinity^34^. On the other hand, peptides have emerged as a powerful tool for cell targeting because they are small, can be manufactured commercially and can be modified conveniently for individual conjugation strategies. Using a phage display approach, specific peptides have been identified that bind to cancer cells^35^, neutrophils^36^ or synovial cells^37^. A potential limitation of using short 7-mer peptides as ligands is that their binding affinity seldom matches that of natural antibodies. However, the limited affinity of individual peptides can be offset by arraying multiple 7-mers together in clusters to increase avidity. Indeed, biopanning via phage display benefits from the avidity that results from five copies of the phage-displayed peptides attached to the pIII coat protein at the tip of the M13 bacteriophage^38^. This strategy for increasing affinity through avidity has recently been emulated by  assembling up to four peptides in a cluster to mimic the array of peptides displayed by pIII^39^. The strategy for displaying multiple EP9 peptides on the surface of PFC emulsion benefits from similar avidity effects given that they efficiently bind to EpiSCs. Coupling of EP9 to the PFC emulsion does not appear to impair its binding to EpiSCs most likely because the peptide was designed with a C-terminal GGGK-Cys tail for conjugation which results in a peptide orientation comparable to that in the original M13 phage^40^.

In the present study, we have shown that EP9 is suitable to facilitate the specific delivery of a PFC-based ^19^F contrast agent to EpiSCs. MR contrast agents based on ^19^F have received increasing attention recently, since ^19^F has several advantageous properties such as a gyromagnetic ratio close to that of ^1^H (83% of ^1^H) and a natural abundance of 100%^41^. Moreover, ^19^F can be found in biological tissue only in trace amounts^18^ and therefore no ^19^F background can be detected in biological samples. Consequently, ^19^F MRI provides high sensitivity and the accumulation of ^19^F atoms within a cellular system can be visualized with high specificity. Moreover, ^19^F MRI does not interfere with anatomical ^1^H MRI and so, ^1^H/^19^F MRI datasets from the same field of view can be merged to gain information about the precise spatial localization of the ^19^F signal. Finally, there is a linear correlation of the ^19^F signal intensity with the concentration of deposited ^19^F atoms which allows for absolute quantification^42^. A class of molecules that carries a high payload of ^19^F atoms are perfluorocarbons, which are fluorinated organic molecules that are neither hydrophilic nor lipophilic and therefore must be emulsified with lipids for utilization in biological systems^43^. In the past, it has been shown that intravenous injection of PFCs results in cellular uptake by circulating monocytes and macrophages that leads to an accumulation of ^19^F atoms at sites of inflammation^19^. This technology has been successfully used for imaging a variety of different inflammatory diseases like myocardial infarction, myocarditis, transplant rejection and pneumonia^21^. To further enhance the specificity of PFCs, and to expand the repertoire of target cells and structures we have recently established a platform to functionalize PFCs with specific ligands^30,32,44^. Using this platform, we have visualized thrombi with ^1^H/^19^F MRI, employing FXIIIa-specific short peptides for delivery of ^19^F-based contrast agents^30^. The absence of any confounding background signal and consequent unequivocal assignment makes ^19^F MRI an attractive modality for tracking specific cells and molecules*.*

Intravenous administration of conventional PFCs results in the fast clearance of PFCs from the blood due to rapid uptake by circulating monocytes and cells of the reticuloendothelial system. In a previous study, we showed that in addition to monocytes and macrophages, EpiSCs also take up PFCs after myocardial infarction in vivo^5^. However, this effect became only visible 3 days after infarction, which was due to: (i) the strong cellular uptake of PFCs by monocytes and macrophages, (ii) their time course of infiltration into infarcted hearts, and (iii) the epicardial layer only reaching its maximum thickness 3 days after MI^4^. To be able to exclusively target EpiSCs—and in particular the early phase of EpiSC activation—it is important to conjugate a EpiSC-specific targeting ligand to the nanoparticle surface and reduce the uptake of PFCs by monocytes/macrophages to the extent possible.

In the present study, we have implemented a two-step strategy in which phage were first positively selected on EpiSCs and then subjected to a second step of negative selection against cellular components of the blood. To further enhance the specificity of the ^EP9^PFC targeting approach, we conjugated EP9 to a PEGylated PFC emulsion. Whereas PEGylation impairs the uptake by monocytes^29,30,45^ subsequent addition of EP9 facilitated efficient uptake by EpiSCs which outcompeted the cellular uptake by highly phagocytic monocytes and therefore enabled cell-specific uptake of ^EP9^PFCs by EpiSCs.

Although we have shown in this proof of principle study that EP9 can be used to target an ^19^F contrast agent to EpiSCs, future use of EPs may include coupling them to tracers for other imaging technologies such as PET, fluorescence-based methods, or ultrasound. Moreover, EPs may be used to enhance the specificity of drug delivery systems. In the past, liposomes have been used as a platform for the delivery of therapeutic drugs^46^. To enhance therapeutic effects and reduce off-target side effects, active targeting of drugs to tissues of interest has been investigated in the tumor field. Liposome formulations for cancer therapy have already been approved clinically^47^. Of note, targeted delivery of antibody-^48^ and peptide-conjugated^49^ drugs has also been used to reduce the adverse consequences of myocardial infarction. As another potential therapeutic option after myocardial infarction, systemic application of thymosin-β4 has been shown to enhance the differentiation of WT-1^+^ lineage-traced epicardial cells into cardiomyocytes^50^. However, the number of newly formed cardiomyocytes was rather low^50^. Promising pharmacological results have also been observed for cardiac fibroblasts which share differentiation pathways in common with epicardial cells. Cocktails of small molecules have been shown to convert fibroblasts into cardiomyocytes^51^, but the reported rate of in vivo conversion of fibroblasts into cardiomyocytes is again rather low^52^. Targeted delivery of pharmacologically active molecules to EpiSCs (using EP9) or to cardiac fibroblasts could result in higher local concentrations thereby augmenting the cardiac regenerative response. Of note, the post-MI epicardium is predisposed for targeting by the newly developed nanoemulsion delivery system, since its vascular endothelium is fenestrated and therefore permits passage of PFCs from the blood stream to epicardial cells^5^.

One important aspect of the targeting of ^EP9^PFCs is the overall biodistribution. In general, unmodified PFCs have a size of 100–200 nm and strongly accumulate in organs of the reticuloendothelial system like the liver and the spleen^44,53,54^. To a lesser extent, PFCs do also accumulate in the kidneys, the bone marrow and the lymph nodes^19^. However, under non-inflammatory conditions PFCs do not enrich in the brain, the heart, the lung, skeletal muscle, eyes or the brain since there is no major influx of inflammatory cells or the presence of a leaky endothelium which allows for the passage of cells or free PFCs. Recently, we have analyzed the biodistribution of GFP-labelled targeting PFCs (^GFP^PFCs)^44^ in mice. Upon intravenous injection, there was a rapid decay of ^GFP^PFCs in blood within 24 h, paralleled by a strong accumulation in the liver and to lesser extend in the spleen. With a hydrodynamic diameter of 200 nm, ^EP9^PFCs have twice the size of ^GFP^PFCs (100 nm). Since it is known that larger size of PFCs reduces the blood half-life but increased accumulation in liver and spleen^54^, we assume that ^EP9^PFCs will display a shorter blood half-life than ^GFP^PFCs and rapidly accumulate in liver and spleen. However, a detailed analysis of the impact of the EP9 peptide on the time course of the biodistribution will be performed in future in vivo studies. Another important aspect for targeting ligands are potential adverse effects. We did not find any evidence for acute cellular cytotoxicity for EP9 or ^EP9^PFCs which is in good agreement with previous preclinical and in vitro studies^19,54,55^. In humans, minor flu-like symptoms had been observed after intravenous administration of PFCs which quickly resolved within 24 h and were attributed to activation of macrophages of the reticuloendothelial system^56^.

Despite all its advantages there are certain limitations when using EP9 as a peptide ligand for targeting EpiSCs. Although EP9 was identified as ligand that bound to EpiSCs but not to circulating immune cells, we cannot rule out the possibility that EP9 may also bind to other cell types within the infarcted heart such as vascular smooth muscle, coronary endothelium, pericytes or fibroblasts. Furthermore, EpiSCs are not a homogeneous group of cells but instead consist of several subtypes. Therefore, it is currently unclear if EP9 binds equally to all cell types within the epicardial layer. Another important aspect is that we observed a decay of the cellular labeling efficacy of EP9 and ^EP9^PFCs upon storage for 24 h, which has to be considered for EP9-mediated targeting approaches. This, could be due to conformational changes or chemical modifications like oxidation of a thioether to sulfoxides on methionine residues. Loss of targeting efficacy after 24 h has no major impact on the targeting of EpiSC since EP9/^EP9^PFCs rapidly bind to EpiSC within minutes. Therefore, ^EP9^PFCs or other EP9-conjugates should be freshly prepared before use or alternatively have to be frozen for long term storage. Additionally, under in vivo conditions the targeting capabilities of EP9 could be further impaired by proteolysis or adhesion of certain serum proteins. However, these important issues can only be resolved in the future by further detailed in vitro and in vivo studies and some of the limitation could be overcome by peptide optimization strategies.

## Supplementary information

Supplementary Information.
