# Supplementary material for: MRI-based molecular imaging of epicardium-derived stromal cells (EpiSC) by peptide-mediated active targeting
Source: Sci Rep. 2020 Dec 10;10:21669. doi: 10.1038/s41598-020-78600-y (PMC7728754; doi:10.1038/s41598-020-78600-y)
Supplement: Supplementary file 1 — Supplementary Information. [file 41598_2020_78600_MOESM1_ESM.pdf]

MRI-based molecular imaging of epicardium-derived stromal cells (EpiSC) by peptide-mediated active targeting

Authors:

Tamara Straub[1], Julia Nave[1], Pascal Bouvain[1], Mohammad Akbarzadeh[2], Siva Sai Krishna Dasa[4], Julia Kistner[1], Zhaoping Ding[1], Aseel Marzoq[1], Stefanie Stepanow[3], Katrin Becker[1], Julia Hesse[1], Karl Köhrer[3], Ulrich Flögel[1], Mohammad R. Ahmadian[2], Brent A. French[5], Jürgen Schrader[1*] and Sebastian Temme[#1*]

Affiliations:

[1]Department of Molecular Cardiology, Heinrich Heine University Düsseldorf, Düsseldorf, NRW, Germany

[2]Department of Biochemistry and Molecular Biology II, Heinrich Heine University Düsseldorf, Düsseldorf, NRW, Germany

[3] Biological and Medical Research Center (BMFZ), Genomics and Transcriptomics Laboratory, Heinrich Heine University Düsseldorf, Düsseldorf, NRW, Germany

[4]Robert M. Berne Cardiovascular Research Center, University of Virginia, Charlottesville, VA, USA

[5]Department of Biomedical Engineering, University of Virginia, Charlottesville, VA, USA

* Shared senior authorship

SUPPLEMENTAL MATERIAL

Figure S1:

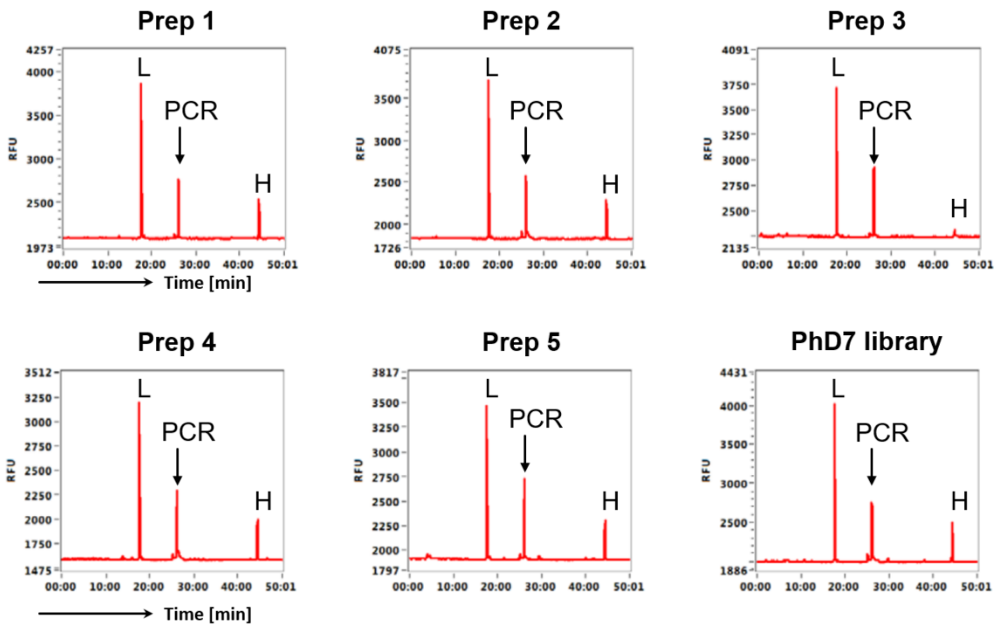

Figure S1: Fragment analysis of PCR products for deep sequencing analysis. Phage of a PhD7 library were selected positively on five individual EpiSC preparations. In a second step, the EpiSC-binding phage were panned against rat blood to remove phage that bind to cellular components of rat blood. The nucleotide sequence of the peptide-coding region of the isolated phage pools as well as the parent PhD7 library as control was PCR amplified and subjected to fragment analysis. The fluorescence signal (RFU) of the PCR products is plotted against the running time (min). The graphs show a strong peak after 18 min for the low base-pair (bp) marker (L), a single sharp peak after 25 min for the PCR products of the phage pools (PCR) and a smaller peak after 43 min for the upper bp marker (H).

Figure S2:

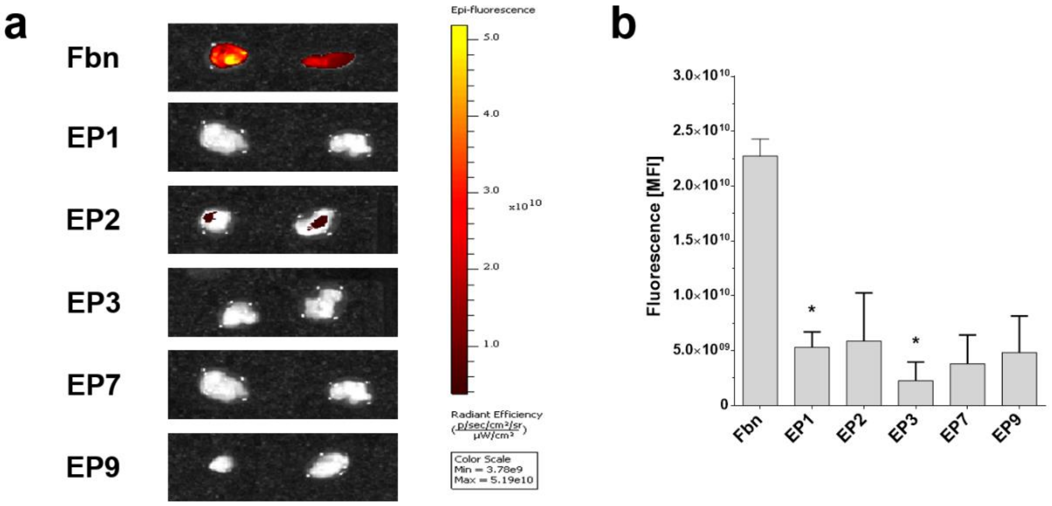

Figure S2: (a) EPs and a fibrin-binding peptide (Fbn) were incubated on *ex vivo* generated thrombi and the binding was determined by IVIS fluorescence analysis. Fbn is based on the MR contrast agent EP2104-R[25] and therefore represents a positive control. The image shows an overlay of the photograph (greyscale) with the carboxyfluorescein-signal of the labelled peptides (hot iron). **(b)** Quantification of the fluorescence signal from (a). Data are mean values ± SD of n = 3 individual experiments.

Figure S3:

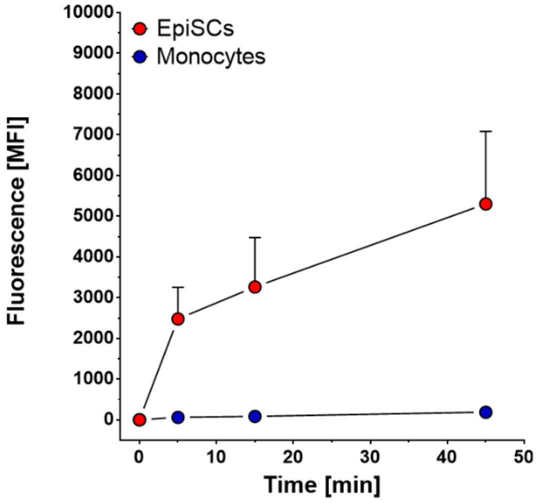

Figure S3: Comparison of the cellular association of EP9 by EpiSC and human primary human monocytes. Cells were incubated with EP9 over a period of 45 minutes at 37 °C. At distinct time points (0, 5, 15 and 45 min), cells were analysed by flow cytometry. Data show the mean fluorescence intensity (MFI) - of the carboxyfluorescein conjugated to EP9 - ± SD of n = 4.

Figure S4:

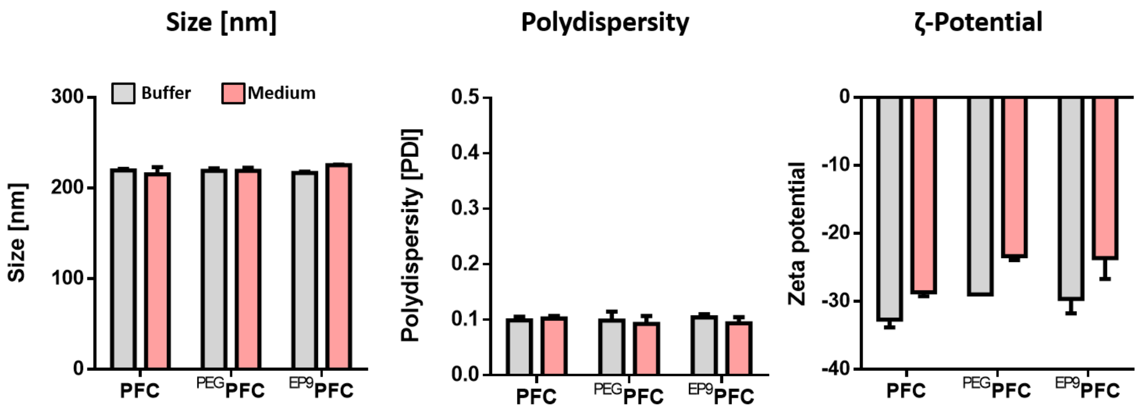

Figure S4: ^{EP9}PFCs, ^{PEG}PFCs and unmodified PFCs were analyzed by dynamic light scattering to determine size, size distribution, and the ζ-potential. Data are mean values ± n=3 independent experiments.

Figure S5:

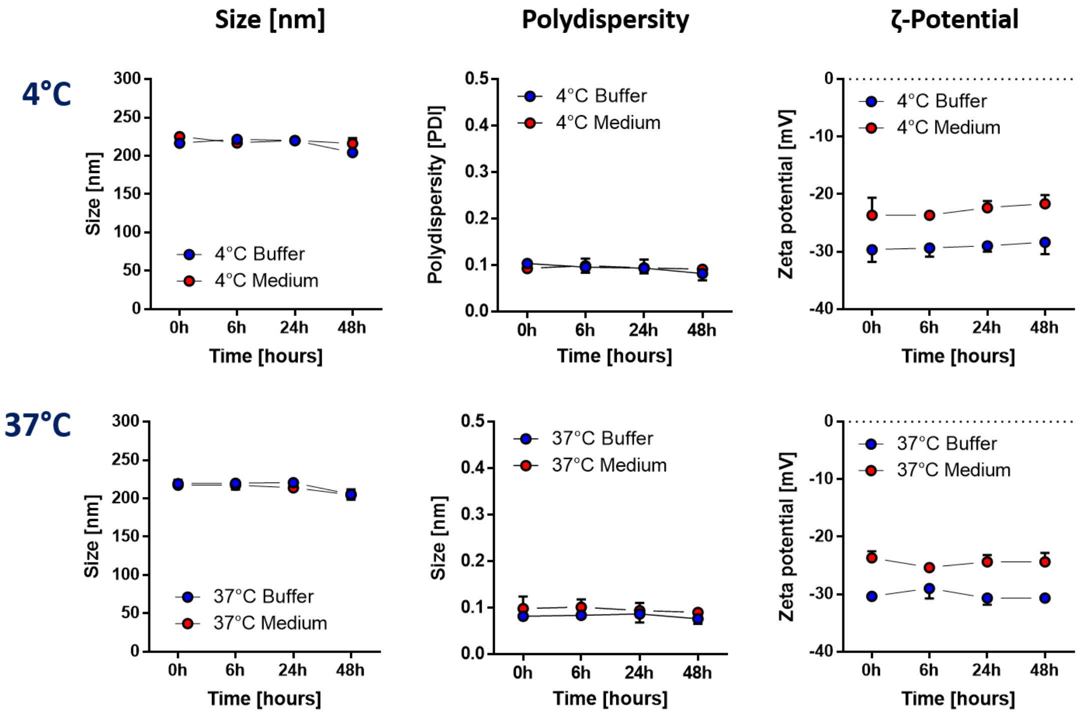

Figure S5: Stability of EP9PFCs: EP9PFCs were suspended in phosphate buffer (buffer, blue) or cell culture medium containing 10% fetal calf serum (medium, red) and incubated for 6 h, 24 h and 48 h. EP9PFCs were analyzed by dynamic light scattering to determine size, size distribution (PDI) and the ζ-potential. Data are mean values ± SD of n=3 individual experiments.

Figure S6:

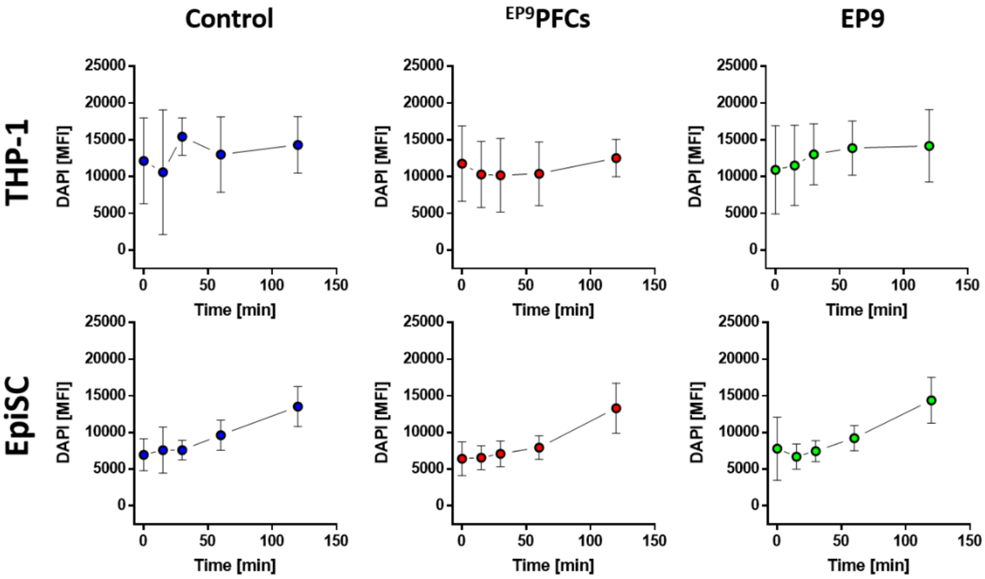

Figure S6: EpiSC and THP-1 were treated with EP9PFCs (red, middle), EP9 peptide (green, right) or left untreated as control (blue, left) for 2 h. Samples were withdrawn at distinct time points and stained with DAPI to label dead cells. The graphs show the mean fluorescence intensity (MFI) of the DAPI signal. Data are mean values ± SD for n=3 experiments.

Figure S7:

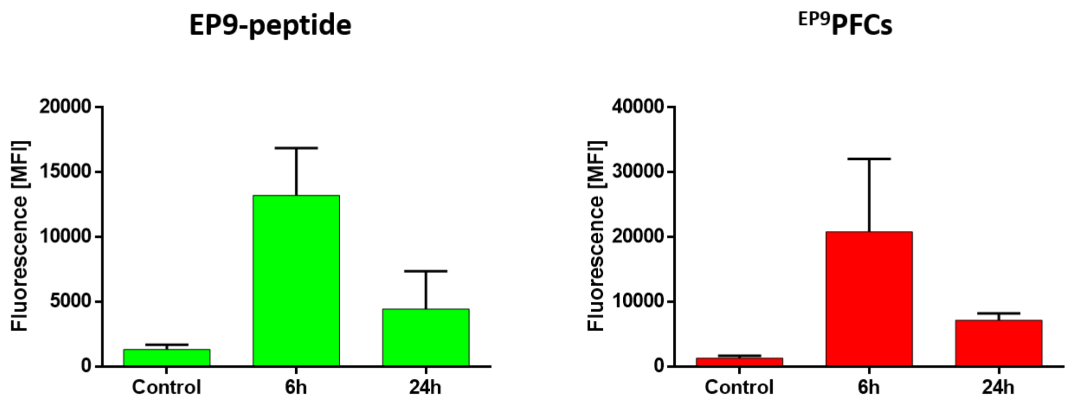

Figure S7: ^{EP9}PFCs (left) and EP9 (right) were stored in phosphate buffer for 0 h, 6 h and 24 h and subsequently incubated with EpiSC. Cells were analyzed by flow cytometry and the mean fluorescence value (carboxyfluorescein) was analyzed. Untreated cells served as background control. Data are mean values of n=3 ± SD.

Table S1: Primers for the deep sequencing

Red: Illumina-sequence; blue: Barcode-sequence; violet: Barcode-adapter; green: Primer-sequence; black: Trp-sequence (for purification). Primers were obtained from BioSpring (Frankfurt am Main, Germany).

Primer Name	Sequence
Ada1-fwd	5'cca tct cat ccc tgc gtg tct ccg act cag cta agg taa cga ttc gca att cct tta gtg gta cct 3'
Ada2-fwd	5'cca tct cat ccc tgc gtg tct ccg act cag taa gga gaa cga ttc gca att cct tta gtg gta cct 3'
Ada3-fwd	5'cca tct cat ccc tgc gtg tct ccg act cag aag agg att cga ttc gca att cct tta gtg gta cct 3'
Ada4-fwd	5'cca tct cat ccc tgc gtg tct ccg act cag tac caa gat cga ttc gca att cct tta gtg gta cct 3'
Ada5-fwd	5'cca tct cat ccc tgc gtg tct ccg act cag cag aag gaa cga ttc gca att cct tta gtg gta cct 3'
Ada6-fwd	5'cca tct cat ccc tgc gtg tct ccg act cag ctg caa gtt cga ttc gca att cct tta gtg gta cct 3'
Ada-Trp-rev	5'cct ctc tat ggg cag tcg gtg atg tct ttc cag acg tta gta aat gaa 3'

Table S2: Enrichment of EpiSC specific peptide sequences (EP)

Relative increase of the 50 most abundant peptide sequences identified by NGS of phage eluates of five different EpiSC isolates (Prep) in relation to the parent PhD7 phage library. The order of the listed EPs (EP1-EP50) represents their mean relative enrichment. The peptide sequences that were selected for binding studies are marked in red.

Number	Sequence	Prep 1	Prep 2	Prep 3	Prep 4	Prep 5	Mean	SD
**EP1**	**SEPIVPL**	**123.1**	**191.2**	**0.1**	**120.8**	**92.2**	**105.5**	**69.2**
**EP2**	**ATKTIAP**	**127.8**	**87.3**	**120.1**	**64.3**	**125.0**	**104.9**	**27.9**
**EP3**	**THVYRDE**	**151.8**	**79.4**	**58.7**	**90.4**	**42.4**	**84.5**	**41.9**
EP4	GSSLTRP	0.1	236.5	0.1	0.1	185.2	84.4	116.
EP5	HLSMAVQ	121.3	0.1	0.1	93.0	142.1	71.3	67.3
EP6	GALAKDE	53.7	91.6	149.1	0.1	33.5	65.6	57.3
**EP7**	**QSHALMA**	**54.6**	**67.2**	**72.2**	**48.7**	**74.4**	**63.4**	**11.3**
EP8	NLKWPTL	60.2	53.2	33.1	0.1	101.9	49.7	37.4
**EP9**	**KLMLPRP**	**47.2**	**56.7**	**24.3**	**56.5**	**27.5**	**42.4**	**15.6**
EP10	RAGNLAM	0.1	31.4	102.5	0.1	62.5	39.3	43.8
EP11	WHSLPKP	0.1	117.0	69.5	0.1	0.1	37.3	53.7
EP12	MSQPSYH	18.5	13.1	74.2	46.0	32.7	36.9	24.5
EP13	QSGTYVP	59.3	36.7	29.7	27.8	19.3	34.5	15.2
EP14	RHVEGPV	43.5	43.6	0.1	49.5	35.0	34.3	19.8
EP15	KTPTLAL	0.1	0.1	163.9	0.1	0.1	32.9	73.3
EP16	WVPLNGR	0.1	27.1	60.0	28.7	47.6	32.7	22.8
EP17	EIRTGFD	0.1	43.6	114.0	0.1	0.1	31.6	49.8
EP18	NARPPTS	7.4	18.3	84.3	31.3	14.1	31.1	31.0
EP19	VAPYPRG	31.5	28.8	29.7	15.6	43.9	29.9	10.1
EP20	SDNLRSD	15.7	17.5	30.4	38.2	20.1	24.4	9.6
EP21	DVSYGQI	0.1	119.6	0.1	0.1	0.1	24.0	53.4
EP22	TDMKVSR	33.3	43.6	20.9	0.1	18.6	23.3	16.4
EP23	SLWDSKG	0.1	40.2	0.1	0.1	75.1	23.1	33.9

EP24	WAADIHM	17.6	25.3	17.5	54.7	0.1	23.1	20.0
EP25	STSKDTL	0.1	21.8	66.8	0.1	20.1	21.8	27.2
EP26	NTGGPKM	19.4	0.1	31.0	8.7	47.6	21.4	18.7
EP27	TQMWAMG	66.7	0.1	0.1	0.1	35.0	20.4	30.0
EP28	SSVHPLA	42.6	20.1	20.2	0.1	11.9	19.0	15.6
EP29	SELGLNN	23.1	14.0	19.6	16.5	21.6	19.0	3.7
EP30	VNLNMEL	25.9	33.2	0.1	7.8	23.8	18.2	13.7
EP31	SDLSSTW	29.6	9.6	0.1	0.1	46.9	17.3	20.5
EP32	LLAPPYW	0.1	0.1	0.1	85.1	0.1	17.1	38.0
EP33	AWALPVS	31.5	15.7	0.1	18.2	18.6	16.8	11.2
EP34	VPLSSRA	39.8	29.7	0.1	9.6	4.5	16.7	17.2
EP35	SSNPIYV	0.1	0.1	0.1	54.7	28.3	16.7	24.5
EP36	YLVPEEP	24.1	0.1	19.6	10.4	28.3	16.5	11.3
EP37	NTLGQVS	38.9	16.6	24.3	0.1	0.1	16.0	16.6
EP38	WQKNDAL	33.3	19.2	0.1	10.4	15.6	15.7	12.2
EP39	YLTGIMT	15.7	12.2	13.5	10.4	26.0	15.6	6.1
EP40	VVNMRGG	18.5	7.9	21.6	20.9	8.9	15.5	6.6
EP41	SVDIWTI	13.0	0.1	17.5	13.9	32.0	15.3	11.4
EP42	TLTVYMS	21.3	20.1	19.6	0.1	13.4	14.9	8.8
EP43	GPQLHGW	9.3	22.7	25.0	0.1	15.6	14.5	10.2
EP44	GSKQLEV	37.0	5.2	17.5	0.1	12.6	14.5	14.3
EP45	TIGKFGP	6.5	12.2	29.0	0.1	24.5	14.5	12.1
EP46	VTLNVNI	22.2	10.5	0.1	11.3	28.3	14.5	11.0
EP47	KHLDQNL	39.8	0.1	0.1	32.1	0.1	14.5	19.8
EP48	KPVPTLS	31.5	7.9	0.1	9.6	22.3	14.3	12.5
EP49	ECCVAGT	37.0	5.2	14.8	13.9	0.1	14.2	14.1
EP50	VMNISQT	0.1	22.7	0.1	0.1	47.6	14.1	21.1

Table S3: Comparative analysis of EP-binding to EpiSCs

EP1, EP2, EP3, EP7 and EP9 were incubated on EpiSCs for 120 min at room temperature and the binding was analyzed by flow cytometry. The fluorescence signal of unstained cells served as background control. Data were analyzed by two-way ANOVA and shown is the p-value of n = 5 experiments. Note that the MFI of EP9 (indicated in red) shows the highest level of statistical significance.

120 Min	EP1	EP2	EP3	EP7	EP9
**EP1**					
**EP2**	0.9720				
**EP3**	> 0.9999	0.9296			
**EP7**	0.9995	0.9977	0.9956		
**EP9**	**0.0037**	**0.0394**	**0.0019**	**0.0106**	
**ctrl**	0.6597	0.2121	0.7742	0.4500	< **0.0001**
